# Evaluation of Different Visual Feedback Methods for Brain—Computer Interfaces (BCI) Based on Code-Modulated Visual Evoked Potentials (cVEP)

**DOI:** 10.3390/brainsci14080846

**Published:** 2024-08-22

**Authors:** Milán András Fodor, Hannah Herschel, Atilla Cantürk, Gernot Heisenberg, Ivan Volosyak

**Affiliations:** 1 Faculty of Technology and Bionics, Rhine-Waal University of Applied Sciences, 47533 Kleve, Germany; 2 Institute of Information Science, Technical University of Applied Sciences Cologne, 50678 Cologne, Germany

**Keywords:** brain–computer interface (BCI), BCI speller, code-modulated visual evoked potentials (cVEP), EEG-based BCI, visual evoked potentials (VEP)

## Abstract

Brain–computer interfaces (BCIs) enable direct communication between the brain and external devices using electroencephalography (EEG) signals. BCIs based on code-modulated visual evoked potentials (cVEPs) are based on visual stimuli, thus appropriate visual feedback on the interface is crucial for an effective BCI system. Many previous studies have demonstrated that implementing visual feedback can improve information transfer rate (ITR) and reduce fatigue. This research compares a dynamic interface, where target boxes change their sizes based on detection certainty, with a threshold bar interface in a three-step cVEP speller. In this study, we found that both interfaces perform well, with slight variations in accuracy, ITR, and output characters per minute (OCM). Notably, some participants showed significant performance improvements with the dynamic interface and found it less distracting compared to the threshold bars. These results suggest that while average performance metrics are similar, the dynamic interface can provide significant benefits for certain users. This study underscores the potential for personalized interface choices to enhance BCI user experience and performance. By improving user friendliness, performance, and reducing distraction, dynamic visual feedback could optimize BCI technology for a broader range of users.

## 1. Introduction

A brain–computer interface (BCI) is a technology designed for real-time communication and control, creating a direct link between the human brain and external devices [1]. A BCI system translates the brain signals from a user into a desired output, enabling computer-based communication or the control of external devices. One of the most common techniques for evaluating brain waves is the electroencephalogram (EEG) [2]. Advances in EEG technology have enabled higher temporal and spatial precision in recording brain activities. This has provided researchers with detailed insights into the brain’s electrical activity and facilitated the development of advanced BCI applications [3].

BCI spellers are designed to provide an interface for typing using brain activity [4]. Spellers can be categorized based on their method for letter selection. Some systems display all letters (targets) on the screen at once, while others allow the user to select letters in multiple steps, with fewer targets on the screen at a time. In this study, a three-step speller is used, which only requires four independent visual stimuli. It has been proven to provide a reliable and easy-to-use experience for the vast majority of users compared to those with fewer steps and more targets [5,6].

EEG activities collected from the scalp can be used to derive visual evoked potentials (VEPs) or, more generally, evoked electrophysiological potentials. VEPs provide important diagnostic data on the functional integrity of the visual system. These electrical signals are produced in response to visual stimuli and can be used to interpret the user’s intent, converting it into control signals for a computer or other device [7,8].

A variation in the standard VEP, known as code-modulated visual evoked potentials (cVEPs), employs a pseudorandom code to create various visual stimuli and has gained increased popularity in recent years [9,10,11]. A cVEP is induced when someone responds to such stimuli, and this can be utilized to control a BCI system in a comparatively quick fashion [12,13].

cVEPs can be put to great use in BCI spellers, where the user is provided with a set of flickering targets (letters or collections of letters), each connected to a unique binary code pattern, the so-called m-sequence, that controls whether the stimulus is displayed or not during the actual frame. The user’s brain signals are evaluated in real-time using pre-recorded target-specific EEG templates from a training session for categorization [1,6,14].

When assessing the effectiveness of BCI spellers, the speed and accuracy with which the user types words, and subjective fatigue assessments, are usually considered as the most important metrics. The information transfer rate (ITR), measured in bits per minute (bpm), is typically employed in the evaluation of BCI performance. It is the speed at which information is transferred, defining the main property of every information channel. ITR is influenced by several factors such as classification speed, accuracy, and the number of targets.

Performance improvements can be achieved through advancements in algorithms. For instance, our prior research [15] enhanced the performance of a cVEP BCI system using novel target identification methods, such as dynamic sliding windows and stimulus synchronization. Other routes to increasing performance include making the interface and the selection process more user-friendly and less fatiguing. Variations in visual feedback for selection have shown promise in Steady-State visual evoked potential-based (SSVEP) solutions [16], motor imagery [17], and other modalities [18]. In this context, visual feedback refers to providing real-time, on-screen feedback about the selection process for the user. This could mean any kind of indicator such as a threshold bar [19] that indicates the progress of target selection to the user.

We hypothesize that visual feedback that maintains user focus on the flickering box will result in stronger, more detectable evoked potentials in a cVEP-based speller, as it distracts the user less from the stimulus. For cVEP-based systems, a novel approach to achieve this would be dynamically changing the target and stimulus size on-screen based on the certainty of detecting given cVEPs, providing real-time feedback to the user, while keeping their focus on the stimulus. The potential performance and usability gain is particularly significant because cVEPs can be more challenging to detect and induce more eye fatigue compared to other VEPs, such as the commonly used SSVEPs.

This study aims to evaluate the effectiveness of such visual feedback in cVEP-based systems by implementing a dynamic interface. The target box will increase in size when the user is trying to select it, making it easier to select and providing valuable feedback during the selection progress, as was previously proposed for SSVEP-based BCIs in [16]. Conversely, the other targets that the user is not focusing on will appear smaller. This dynamic interface may be a superior kind of visual feedback solution compared to other methods, which might be more distracting. By allowing the user to select targets more easily, this approach could enhance performance and reduce fatigue as well.

To evaluate the effectiveness of the dynamic interface, we compared it with the more commonly used threshold bar interface element [19,20,21,22]. Participants tried both versions of a three-step-speller cVEP-based BCI system, allowing us to compare their performance. We also gathered feedback on their subjective experiences and preferences, which helped us conclusively assess whether and in what sense the dynamic interface could be superior in cVEP-based BCI systems. Furthermore, we collected information regarding participant fatigue before and after the experiment using a standardized questionnaire. The study can also reinforce the reliability of this type of three-step cVEP speller and its claim of achieving a nearly 100% literacy rate [6]. Additionally, by evaluating the visual feedback solutions, this research seeks to enhance these types of BCI spellers in terms of both performance and user-friendliness, with the overarching goal of making BCI technology accessible and effective for everyone.

In this section, we transitioned from a broad description of brain–computer interfaces to a detailed explanation of the specific BCI system used in this study, emphasizing the significance of visual feedback. In the following section, we describe the user experience of the employed system in all its technical detail, organized based on the user experience timeline.

## 2. Materials and Methods

### 2.1. Participants

A total of 48 participants (30 females, 17 males, and 1 non-binary) took part in this study. The average age of the participants was 23.9 years, with a standard deviation (SD) of ±3.62. All participants provided written consent in adherence to the Declaration of Helsinki, and the study received approval from the ethical committee of the medical faculty at the University of Duisburg-Essen. Participants could withdraw from the experiment at any time without providing any reasons. The collected data were stored anonymously for analysis purposes, ensuring the confidentiality of the participants. Each participant received EUR 20 for their participation in the study.

### 2.2. Experimental Protocol

The experiment was conducted in the BCI-Lab of Rhine-Waal University of Applied Sciences (HSRW). First, participants received an information sheet detailing the nature of the experiment. After providing their personal information and written consent, they completed the pre-questionnaire (Table 1), which included questions about their experience with BCI systems, vision prescriptions, and level of tiredness. Subsequently, the electrode cap was applied, and the participants were further briefed on the procedure and operation of the speller.

Following these explanations, participants engaged in a preliminary practice phase to familiarize themselves with the speller. During this phase, they chose a five-letter word (such as their name) and practiced selecting letters with the speller. The threshold, gaze shift, and time window settings were calibrated as necessary during this initial free-spelling practice phase. Following the free-spelling practice phase, the participants were split based on their number to counter the potential effects of becoming better with experience during the session. Subject number was given in order of attendance.

Odd-numbered participants started with the dynamic interface, while even-numbered participants started with the threshold bar interface. During the spelling session, participants were first instructed to spell the word “BCI”. After this, subjects were again split based on their subject number. Odd-numbered subjects proceeded with spelling “PROGRAM” and then “HAVE_FUN”. Even-numbered subjects did this in the opposite order, spelling “HAVE_FUN” first, then “PROGRAM”. After this first round of three words (plus the free-spelling practice), the subjects switched to the other version and spelled same three words again (in the order described above).

These words for the spelling phase were also selected to maintain a balance in class representation (not including the target with the “UNDO” function). When completing the spelling phase without mistakes, the first target needs to be selected 20 times, the second one 16 times, and the third one 18 times (“HAVE_FUN” and “PROGRAM” are perfectly balanced).

Spelling phases concluded automatically upon correct word spelling. On average, each subject’s spelling session (just spelling) lasted 12 to 15 minutes. Resulting accuracy, ITR, and OCM values were recorded for all completed tasks. After successfully completing the spelling session, participants filled out the post-questionnaire (Table 1), which included questions about their impressions, opinions, and experiences with the BCI system and the two interfaces.

Finally, participants had the opportunity to clean their hair from the conductive gel and received documentation confirming their eligibility for compensation.

### 2.3. Hardware

Due to the high number of participants, the study utilized three Dell Precision Desktops equipped with NVIDIA RTX 3070 graphics cards, running Microsoft Windows 10 (21H2) Education on Intel i9-10900K processors (3.70 GHz). For presenting the stimuli, modern Asus ROG Swift PG258Q displays (Full-HD, 240 Hz maximal vertical refresh rate) were used.

EEG data were collected using g.USBamp amplifiers (g.tec medical engineering GmbH, Schiedlberg, Austria), employing all 16 signal channels. Electrodes were placed according to the international 10-20 system at the following positions: P7, P3, Pz, P4, P8, PO7, PO3, POz, PO4, PO8, O1, Oz, O2, O9, Iz, and O10. The reference electrode was positioned at Cz, and the ground electrode was placed at AFz. During the preparation stage, regular abrasive electrolytic electrode gel was applied between the electrodes and the scalp to reduce impedances to less than 5 kΩ.

### 2.4. GUI

**Figure 1:**
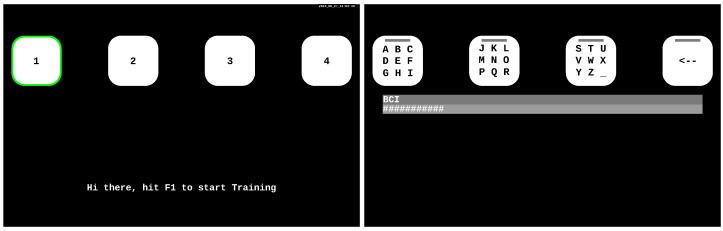
(**Left**) The GUI screen representing the start of the training phase, where the 1st target is marked with a green frame. This mark changes to the consecutive targets during the training phase. (**Right**) The GUI speller screen during the BCI spelling phase with the threshold bar interface. Letter selection required three steps: to type, e.g., the letter B, the participant first selects the ‘A–I’ box (1st target), followed by the ‘A–C’ box (2nd target), and finally the B box.

The graphical user interface (GUI) presents four selection options, as illustrated in Figure 1.

The GUI spelling software was organized as three-step speller following its successful utilization in previous research [6,23]. It includes 26 letters and one underscore character (used instead of a space), divided into three boxes. For example, to spell the letter “A,” the user first selects the group “A–I” in the first step. Then, they select the group “A–C” in the second step, and finally, the individual letters “A,” “B,” and “C” are presented, allowing the user to select the desired letter “A” in the final third step.

### 2.5. Training

During the recording phase, four stimuli were observed sequentially from 1 to 4 by the participants, as illustrated in Figure 1(Left). The recording was grouped into six blocks of training, denoted as $n_{b}=6$. Within each block, every stimulus was focused on once, resulting in a total of 6×4=24 trials. Each trial lasted for 2.1 seconds, during which the code pattern was displayed for two cycles.

A visual cue, represented by a green frame, indicated the specific box towards which participants were required to direct their gaze. Following each trial (gazing on a target), the subsequent target the user needed to focus on was highlighted, and the flickering paused for one second. After completing a block (all four targets), the software transitioned to the next block of training, with a one-second pause until a total of 6×4=24 trials were accomplished. Once the training phase was completed, the spelling tasks began.

### 2.6. Spelling Phase

In the spelling exercise, four boxes were displayed on the screen. As indicated in Figure 1(Right), the first 3 boxes from left to right contain letters, and the fourth is the “UNDO” function, which is used as either a backspace or delete button (when going back is not an option, the last spelled letter is deleted). In copy-spelling mode, the box turns green when the correct box is selected, and red when the wrong one is selected. The participants spelled the words as outlined in the experiment protocol (see Section 2.2). The underscore stands in for the *space* character. Errors are corrected using the UNDO feature of the spelling interface.

### 2.7. Visual Feedback

**Figure 2:**
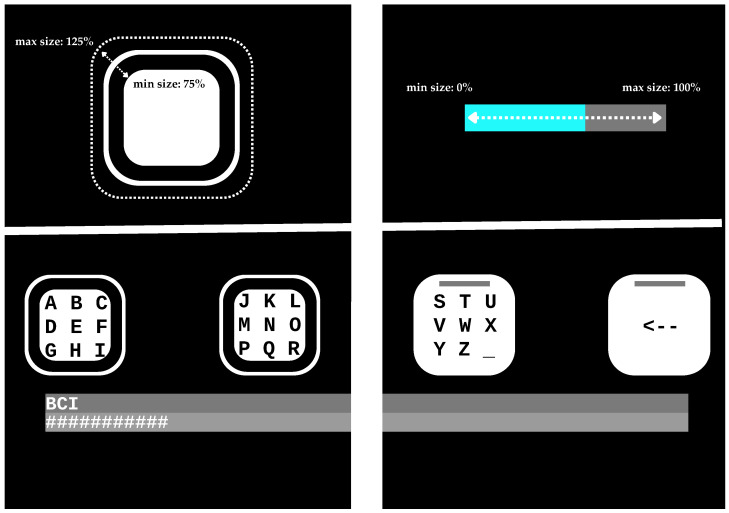
The screenshots of the starting state and during the spelling of both types of visual feedback: the dynamic interface on the left and the threshold bar interface on the right. (**Top left**, going from the outside to the inside) Showcasing all limits of the box: the maximum size (125%), which appears when the certainty is over the 75% of the threshold, the normal (100%) outline of the box, and the minimum size, which is shown when the certainty is below 10% of the threshold. (**Top right**) Showcasing the threshold bar interface, changing in size depending on the certainty, reaching 100% when the threshold is reached. (**Bottom left**) A cut-out of the GUI screenshot with the dynamic interface, showing the starting state before the spelling task begins. (**Bottom right**) A cut-out of the GUI screenshot of the threshold bar interface, showing the starting state before the spelling task begins.

The visual feedback element in this study was either the threshold bar or the dynamic interface. These elements are shown at the top of Figure 2. At the bottom of the figure, two half GUI screenshots are presented side by side, taken at the start of the spelling phase. These screenshots showcase the initial state of the visual feedback elements.

At the top of Figure 2, the dynamic size change in the target box is illustrated. The size depends on how close the certainty is (ΔC) (the calculation of certainty is explained in Section 2.9) value is to the threshold (β). When the certainty reaches the chosen threshold value (e.g., 0.15), the classification is complete, and the respective box will be selected. The size is kept at a minimum (75% size) when the certainty is below 10% of the set threshold value and at a maximum size when it is above 75% of the set threshold value. This means that the target box changes its size dynamically between 75% and 125% of the original size when the certainty is between 10% and 75% of the set certainty value threshold.

Figure 2 also showcases the threshold bar visual feedback element, which we used as a reference to evaluate the performance of the dynamic version. This visual feedback was developed and utilized in previous cVEP systems by Volosyak et al. (e.g., [6]). A bluish bar grows in size from 0% (left) to 100% (right) as the certainty (ΔC) reaches the set threshold value.

### 2.8. Stimulus Presentation

The spelling interface (utilizing the system most recently used in [20]) incorporates four distinct stimulus options, arranged as a 1 × 4 matrix of boxes, meaning that the number of targets (K) was four. These are 282 × 282 pixels by default in this experimental setup (see Figure 1(left)). The cVEP stimuli code (c) was a 63-bit m-sequence [24], where “0” represents “black” and “1” represents “white,” showcasing a state of complete contrast. The remaining stimuli, $c_{k}$ (for k=2,…,K where K=4), were generated by circularly left (or right) shifting $c_{1}$ by 4, 8, or 16 bits.

(1) $$c_{1}=101011001101110110100100111000101111001010001100001000001111110$$

The duration of a stimulus cycle in seconds can be calculated by dividing the code length by the monitor refresh rate *r* in Hz; in this experiment, 63/60 = 1.05 s. The refresh rate used was 240 Hz, so the stimulus changed in accordance with the bit sequence, but for every fourth frame.

Spatial filters were developed using the information gathered during the recording phase for classification. Canonical correlation analysis (CCA) [25] was used on the training trials for this purpose. CCA is a statistical method used to analyze the relationship between two multi-dimensional variables by finding linear combinations that maximize their correlation.

### 2.9. Classification

Following the methodology outlined in [6], CCA can be applied to two multi-dimensional variables $X\in R^{p\times s}$ and $Y\in R^{q\times s}$ to analyze their relationship. CCA searches for the weights $w_{X}\in R^{p}$ and $w_{Y}\in R^{q}$ that maximize the correlation, ρ, between $x=Xw_{X}$ and $y=Yw_{Y}$ by solving:

(2) $$\rho \left(x,y\right)=\frac{w_{X}^{T}XY^{T}w_{Y}}{\sqrt{w_{X}^{T}XX^{T}w_{X}}\sqrt{w_{Y}^{T}YY^{T}w_{Y}}}.$$

Here, the CCA weights, used as a spatial filter in the online spelling, were constructed as follows. Each training trial was stored as an m×n matrix, where *m* denotes the number of signal channels (here m=16) and *n* denotes the number of samples (here, three 1.05 s stimulus cycles with a 600 Hz sampling frequency , i.e., n=1.05·600·3=1890). In total (given 6 training blocks ($n_{b}$), 24 such trials $T_{i}^{j}\in R^{m\times n},i=1,\ldots ,K,j=1,\ldots ,n_{b}$ were recorded.

For each target, individual templates $X_{i}\in R^{m\times n}$ and filters $w_{i}$ were determined (i=1,…,K). For the generation of spatial filters, the two matrices were constructed:

(3) $${\bar{X}}_{i}=\frac{1}{n_{b}}\sum_{j=1}^{n_{b}}T_{i}^{j}\;\mathrm{and}\;\bar{X}=\left[{\bar{X}}_{1}|{\bar{X}}_{2}|\cdots |{\bar{X}}_{K}\right].$$

The online classification was performed if a new data block was added to a data buffer $Y\in R^{m\times n_{y}}$ with dynamically increasing samples.

For target identification, the data buffer Y was compared to the reference signals $R_{i}\in R^{m\times n_{y}},i=1,\ldots ,K$, which were constructed as a sub-matrix of the corresponding template $X_{i}$.

Correlations $\lambda_{k}$ between the reference signals and the data buffer were calculated as follows:

(4) $$\lambda_{k}=\frac{Yw_{k}}{R_{k}w_{k}},\;k=1,\ldots ,K.$$

The classifier output *C* was then determined as follows:

(5) $$C=\arg\max_{k}\lambda_{k},\;k=1,\ldots ,K.$$

A sliding window mechanism was implemented for online spelling. BCI outputs were only performed if a threshold criterion was met. The EEG amplifier transferred data blocks in chunks of 30 samples (every 0.05 s, as the sampling rate was set to 600 Hz). For the sliding window mechanism, it was required that the number of samples per block is a divider of the cycle length.

The data buffer Y was updated dynamically with each new data block, incrementing $n_{y}$ by 30 samples as long as $n_{y}<n$. The certainty, ΔC, was defined as the distance between the highest and second highest correlation needed to surpass a threshold value, β, which was set to 0.15. For some participants, β was adjusted during the familiarization run to avoid misclassification.

BCI outputs were generated only if ΔC>β. When this condition was met, the data buffer Y was cleared, followed by a specified, usually two-second gaze-shifting phase, during which data collection and visual stimulation were paused, allowing the users to shift their gaze to another target.

The minimum window length and the gaze-shifting period limited the highest achievable information transfer rate of the BCI speller. Individual adjustments to the time window length were made to optimize performance, resulting in varying maximum possible ITRs among participants.

### 2.10. Measures of the BCI Performance

The BCI system’s performance was evaluated using commonly used accuracy (Acc.), the information transfer rate (ITR), and in the form of the output characters per minute (OCM).

Accuracy: The accuracy was calculated by dividing the total number of correct selections (classification steps necessary for word completions were considered single commands), including user-necessary corrections during speller execution, by the overall commands classified. The resulting accuracy value was displayed as a percentage value on the speller interface.

OCM The output characters per minute (OCM) measures typing speed by dividing the total number of output characters by the time taken to type them. OCM accounts for the error correction time, as the participants will require additional time for corrections if mistakes are made.

ITR The information transfer rate (ITR) was calculated in bits per minute (bits/min) using the following formula:

(6) $$B=\log_{2}N+P\log_{2}P+\left(1-P\right)\log_{2}\left[\frac{1-P}{N-1}\right],$$

where

*B* = information transferred in bits;
*N* = number of targets (for this study it is equal to 4);
*P* = classification accuracy.

To obtain the ITR in bits/min, *B* is multiplied by the average classification time in minutes. For more information and tools to calculate ITR, visit our webpage: https://bci-lab.hochschule-rhein-waal.de/en/itr.html (accessed on 23 June 2024).

### 2.11. Questionnaire

A questionnaire was designed to collect participant feedback, with sections dedicated to both pre-experiment and post-experiment questions. These sections were intended to be completed respectively before and after the experiment, focusing on assessing general user experience as well as feedback regarding the two interfaces used. For further information, refer to Table 1, which outlines these pre- and post-experiment questions.

## 3. Results

All statistical analyses were performed using JAMOVI software (The jamovi project, version 2.3, 2022) and Microsoft Excel (version 2021, build 2406). The spelling exercise was successfully completed by every subject, except Subject 27, who reported “too much dizziness”; thus, they did not finish the last spelling task. On average, participants achieved an accuracy of 95.57 % with a standard deviation (SD) of 6.12, and an ITR of 53.55 bits/min (SD: 16.25), resulting in an OCM of 9.24 (SD: 2.56).

The results corresponding to each subject are presented in Table 2. The table illustrates the average accuracy, ITR, and OCM values of both the interfaces together and when separated.

Results per task are represented in Table 3, where the performance value averages (time, accuracy, ITR, OCM) are presented in a study-wide and per interface split manner for each spelling task.

The average and per task performance was very similar among the interfaces. With the threshold bar interface, participants reached an average of 95.71% accuracy with an SD of 5.91, an average ITR of 54.58 with an SD of 15.49, and an average OCM of 9.41 with an SD of 2.47.

With the dynamic interface, participants reached an average of 95.81% accuracy with a standard deviation (SD) of 6.32, an average ITR of 52.34 with an SD of 16.91, and an average OCM of 9.04 with an SD of 2.64.

### Evaluation of the Questionnaires

Among the participants, 32 had never used a BCI system before, while 13 had prior experience (with three missing values). A total of 26 participants did not require vision correction, while 18 wore their vision correction, and 4 did not use their vision correction during the experiment. On average, participants slept 7.14 hours (SD = 1.08) the night before the experiment.

To assess changes in tiredness levels due to the BCI speller usage, the participants rated their tiredness before and after the experiment on a scale from 1 (not at all) to 6 (very much). The participants reported similar levels of tiredness before the experiment (*M* = 2.44, SD = 1.07) compared to after the experiment (*M* = 2.50, SD = 1.13). A Wilcoxon signed-rank test revealed that there was no statistically significant difference in reported levels of tiredness (*W* = 117, *p* = 0.249). Following the experiment, participants rated how disturbed they were by the flickering and how easy it was to concentrate on the boxes, again on a scale from 1 (not at all) to 6 (very much). The average ratings were *M* = 2.60 (SD = 1.27) for disturbance and *M* = 4.13 (SD = 1.75) for concentration ease. Regarding the reliability of the BCI as a control method, 32 participants answered “Yes”, indicating that they consider the BCI to be a reliable control method, 15 answered “Maybe” and only 1 participant selected “No”. When asked if they could use the system daily, 21 participants chose “Maybe”, 19 selected “Yes”, and 5 chose “No” (with three missing values). Furthermore, on average, the participants indicated that the system can be used for 1.46 h without any breaks. Most participants (44) indicated they would repeat the experiment, while only two answered “Maybe” (one missing value).

**Figure 3:**
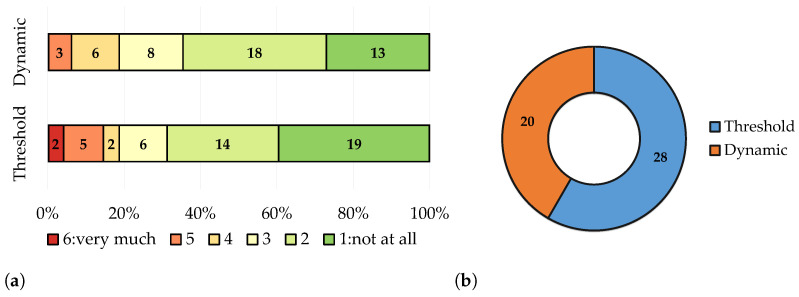
Participants ’ questionnaire responses regarding the two speller interfaces. (**a**) Percentage distribution of the selected answer for the question “To what extent did you find the interface distracting?”. Ratings ranged from 1 (not at all) to 6 (very much). (**b**) Pie-chart of participants selected interface preference.

To evaluate preference differences between the speller interfaces, the participants were asked to what extent they perceived either version as distracting, once more on a scale from 1 (not at all) to 6 (very much). The average rating for the threshold bar interface was *M* = 2.29 (SD = 1.50), while for the dynamic interface it was *M* = 2.33 (SD = 1.19). A Wilcoxon signed-rank test indicated no statistically significant difference between the two interfaces in terms of perceived distraction (*W* = 317, *p* = 0.798). Finally, the participants indicated their preferred speller interface, with 28 preferring the threshold bar interface and 20 preferring the dynamic interface. A binomial test showed that these proportions did not significantly differ from an equal preference assumption. A visual representation of the results concerning the speller interfaces is provided in Figure 3.

## 4. Discussion

Achieving an average accuracy of 95.57% and an ITR of 53.55 bits/min with a 3-step speller highlights the peak performance within this category of spellers. [20,26]. Additionally, the lowest average accuracy recorded was 87.17%, which is decisively above the commonly established 70% accuracy mark for BCI literacy [6]. These findings emphasize that modern cVEP-based BCIs are capable of successful operation across a diverse population.

The primary goal of this study was to compare the performance of cVEP-based BCIs using two different interface types. On average, both interfaces performed well, with subjects using the threshold bar interface achieving slightly better ITR and OCM, while the dynamic interface slightly improved accuracy. However, these differences were not statistically significant.

### 4.1. Statistical Analysis and Validation

Prior to conducting further analysis, the normality of the data was assessed using the Shapiro–Wilk test, confirming that the average ITR for the threshold bar interface (p=0.6373), average OCM for the threshold bar interface (p=0.4250), average ITR for the dynamic interface (p=0.2567), and average OCM for the dynamic interface (p=0.1728) were normally distributed.

Subsequently, a paired *t*-test was conducted to compare the average ITR and OCM values between the two interfaces. The results indicated that there was no significant difference in average ITR (t(47)=0.188,p=0.188) and average OCM (t(47)=0.173,p=0.173) between the threshold bar and dynamic interfaces.

The following factors (for the questions see Table 1) were also tested and did not have a significant influence on the comparative results of the interfaces: participants’ age, whether the interface was used in the first or second round, and past BCI experience. The performance metric averages stayed very similar, no matter the grouping.

### 4.2. Top Quartile Performance Analysis

Even the smallest difference disappears when we examine the top quartile of performances (top 25%, top 36 spelling tasks). Table 4 shows how the average performances of the top quartile are nearly identical, regardless of the interface used. This suggests that neither interface significantly contributed to achieving outstanding performance overall. Consequently, we need to explore potential subject-wise differences.

### 4.3. Subject-Wise Performance Comparison

To further examine the potential differences between the two interfaces, we compared the average values and calculated the performance difference between the interfaces for each subject by subtracting the threshold bar’s averages from the dynamic interface averages. The average ITR difference was −2.16 with an SD of 11.18, favoring the threshold bar interface, and the average OCM difference was −0.35 with an SD of 1.77, also slightly in favor of the threshold bar interface. This was expected based on the average performance scores.

However, to identify potential outliers who had drastically better performance with one interface than the other, we calculated the z-scores. This helped identify subjects who had a significantly (p=0.05) better performance with one of the interfaces. Beforehand, we used the Shapiro–Wilk test to confirm that the *p*-values for ITR (*p*-value: 0.2441) and OCM (*p*-value: 0.2162) differences were greater than 0.05, indicating that we fail to reject the null hypothesis that the data come from a normal distribution. Similarly, a Kolmogorov–Smirnov test showed that ITR (*p*-value: 0.6664) and OCM (*p*-value: 0.5083) differences indeed follow a normal distribution.

**Figure 4:**
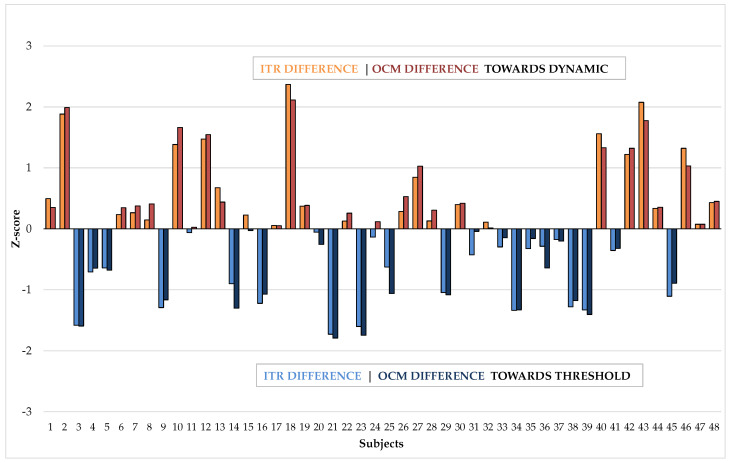
Illustration of z-scores calculated for the performance difference averages. Positive values mean that the dynamic interface performed better, while negative values indicate that the threshold bar interface performed better.

Figure 4 presents all the Z-scores for every subject, illustrating both the average ITR and average OCM differences. As shown in the figure, there are three instances where the 1.95 (p=0.05) mark is reached. Subject 2 had an average OCM difference z-score of 1.99, Subject 18 had an ITR difference z-score of 2.36 and an OCM difference z-score of 2.11, and Subject 43 had an ITR difference z-score of 2.07. Subject 2 experienced a 3.17 increase in OCM. For Subject 18, there was a 24.33 increase in ITR and a 3.39 increase in OCM with the dynamic interface. Subject 43 saw a 21.07 increase in ITR. All of these instances are cases where the subject performed significantly better with the dynamic interface. This leads to the important conclusion that while on average there is no significant difference between the interfaces, 3 out of 48 participants (6.25%) performed significantly better with the dynamic one. This is a crucial finding, especially considering the overarching goal of making BCI available and usable for everyone.

### 4.4. Subject-Wise Perceived Level of Distraction Comparison

Participants on average found the threshold bars more distracting, but not significantly (Figure 3). There were also fewer participants (3 compared to 7) who rated the dynamic interfaces as very/highly distracting (5 and 6 on the Likert scale).

**Figure 5:**
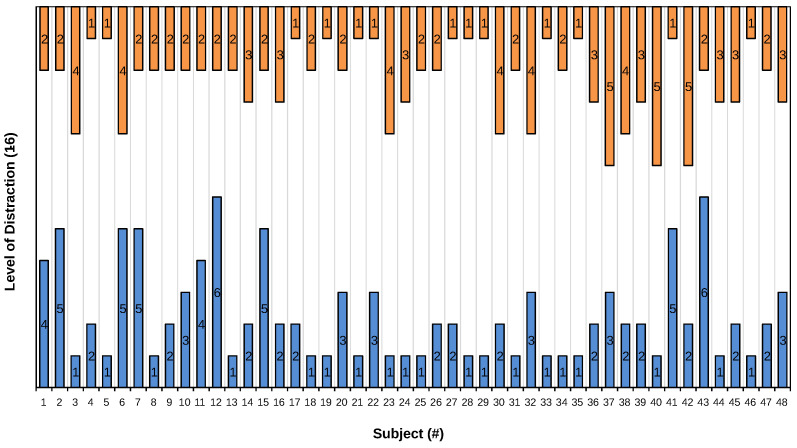
Illustration of the perceived level of distraction. Calculated based on the 6-point Likert scale responses, as the dynamic interface distraction and the threshold bar interface distraction. Orange values refer to the ratings of the dynamic interface, while blue ones to the threshold bar interface.

Examining the differences reveals that there were cases where subjects found one interface significantly more distracting. Figure 5 shows the differences between the two interfaces for each subject. Calculated based on the 6-point Likert scale responses, the difference is obtained by subtracting the threshold bar interface distraction score from the dynamic interface distraction score. The highest, 4-point difference visible on the figure comes out to be exactly the upper and lower bound when a standard Interquartile Range (IQR) method is performed with a multiplier of 1.5 to detect outliers.

Subjects 12, 40, 41, and 43 achieved this 4-point difference. In three cases, the dynamic interface was heavily preferred in terms of perceived distraction, while in one case (Subject 40), the threshold bar interface was deemed a lot less distracting. Importantly, the authors want to highlight the potential error on the part of Subject 40 when answering the related questions. Subject 40 may have mixed up the two interfaces, since contrary to their answer, the subject performed better (+15.27 ITR and +2 OCM) and achieved the highest accuracy difference (+14%) in favor of the dynamic interface.

Either way, these findings further strengthen our conclusion that having a different interface was significantly beneficial for some participants. In this instance, three participants (6.25%) strongly preferred the dynamic interface, while one participant (2.08%) strongly preferred the threshold bar interface.

### 4.5. Conclusion on the Dynamic Interface

We can conclude that while the two interfaces generally achieve a similar performance, there are notable exceptions.

Importantly, there were strong outlier cases where a subject either preferred one interface significantly more in terms of its distraction level or performed significantly better with one interface. Aligning with our hypothesis, this almost exclusively favored the dynamic interface, as evidenced by Figure 4 and Figure 5.

Therefore, the conclusion of the study is that the employed dynamic interface solution can serve as a significant improvement by ensuring that there are less extreme outliers in terms of performance and reducing user distraction without affecting overall average performance.

If implemented as an optional choice, where users are provided with a phase to select the optimal interface for themselves, it has the potential to significantly enhance user experience and performance. This is evidenced by Table 5, which compares the average performance of the current mixed interface setup with the envisioned best-suiting interface setup.

These metrics project the expected performance during further use if the optimal interface is chosen following an objective performance assessment. This also calls for a shorter, more automated interface selection period.

### 4.6. Future Research Directions

Some participants explicitly mentioned during the experiment that they found the threshold bars more “rewarding” or “fun.” This could be a potential area for improvement, possibly through incorporating colors or smoother transitions between sizes, allowing for continuous transitions between different threshold levels.

Future research may focus on experienced users, as even the smallest performance difference disappeared when the we looked at the best performers (see Section 4.2). Additionally, users reported the dynamic interface to be less distracting (see Figure 5). These observations support the initiative of conducting longer sessions to find out if extended use favors the dynamic interface more. Collecting user feedback on perceived levels of eye fatigue after each session, possibly on a larger scale, could provide more detailed insights into differences in eye strain.

Another improvement could be to implement the dynamic interface during training to accommodate potential differences in EEG patterns caused by constant size changes.

Implementing the dynamic interface as an optional feature could be a straightforward improvement for cVEP BCIs. However, this increases the time needed for the system to be ready for use. A potential future direction is to find the quickest way to introduce the subjects to both interfaces and to let them reliably choose their preferred one.

## Figures and Tables

**Table 1:** Used questionnaires with all the questions and answer options.

Pre-Questionnaire
Have you ever used a BCI system? If yes, please add some information about it.
Do you have a vision prescription? If yes, are you wearing a reading aid now?
How tired do you feel right now? 1: not at all, 6: very much
How many hours did you sleep last night?
Post-Questionnaire
How tired do you feel right now? 1: not at all, 6: very much
Did you find the flickering disturbing? 1: not at all, 6: very much
Was it easy for you to concentrate on the boxes? 1: not at all, 6: very much
To what extent did you find the threshold bars distracting? 1: not at all, 6: very much
To what extent did you find the dynamic feedback boxes distracting? 1: not at all, 6: very much
Do you prefer using the threshold bar verion (A) or the dynamic feedback version (B)? A/B
Would you repeat the experiment? Yes/No/Maybe
Could you use the system daily? Yes/No/Maybe
In your opinion, how long can the system be used without breaks?
Do you think the BCI is a reliable control method? Yes/No/Maybe

**Table 2:** The average accuracy, ITR, and OCM values, both with and separately of each of the interfaces. Additionally, the table shows the interface where the users achieved a higher average ITR with and their subjective preference.

	Study-Wide Averages			Threshold bar Interface Averages			Dynamic Interface Averages				
#	ACC	ITR	OCM	ACC	ITR	OCM	ACC	ITR	OCM	BETTER WITH	PREFERENCE
**1**	87.2%	35.15	6.72	81.4%	33.45	6.58	93.0%	36.85	6.85	Dynamic	Dynamic
2	95.2%	62.67	10.74	91.7%	53.22	9.16	98.7%	72.12	12.32	Dynamic	Dynamic
3	88.6%	33.69	5.88	89.2%	43.60	7.47	87.9%	23.78	4.29	Threshold	Threshold
4	99.4%	51.24	8.63	100.0%	56.26	9.38	98.7%	46.22	7.89	Threshold	Threshold
5	100.0%	74.51	12.42	100.0%	79.17	13.20	100.0%	69.85	11.64	Threshold	Threshold
6	97.0%	52.00	8.96	97.4%	51.76	8.83	96.7%	52.23	9.09	Dynamic	Dynamic
7	94.8%	55.82	10.18	94.6%	55.42	10.03	95.0%	56.21	10.34	Dynamic	Dynamic
8	91.6%	50.98	9.21	91.9%	51.24	9.02	91.4%	50.73	9.39	Threshold	Dynamic
9	92.6%	25.01	4.35	100.0%	33.32	5.55	85.2%	16.71	3.14	Threshold	Dynamic
10	95.8%	60.06	10.21	93.9%	53.39	8.92	97.6%	66.73	11.50	Dynamic	Dynamic
11	97.3%	45.45	7.85	97.4%	46.87	8.00	97.3%	44.03	7.70	Threshold	Dynamic
12	92.9%	30.37	5.32	89.4%	23.22	4.13	96.3%	37.53	6.51	Dynamic	Dynamic
13	91.3%	49.30	9.08	90.2%	46.60	8.87	92.4%	52.00	9.29	Dynamic	Threshold
14	87.4%	55.88	10.68	89.9%	61.98	12.01	84.9%	49.77	9.36	Threshold	Threshold
15	97.7%	64.71	10.99	96.7%	64.52	11.19	98.7%	64.90	10.79	Dynamic	Dynamic
16	97.6%	53.20	9.06	100.0%	61.11	10.19	95.1%	45.29	7.94	Threshold	Threshold
17	100.0%	58.20	9.70	100.0%	58.98	9.83	100.0%	57.42	9.57	Threshold	Threshold
18	94.9%	61.51	10.59	89.8%	49.34	8.89	100.0%	73.68	12.28	Dynamic	Threshold
19	100.0%	73.85	12.31	100.0%	72.86	12.14	100.0%	74.84	12.47	Dynamic	Threshold
20	97.0%	51.25	9.00	96.4%	52.64	9.40	97.6%	49.86	8.60	Threshold	Threshold
21	98.1%	55.54	9.29	100.0%	66.29	11.05	96.2%	44.80	7.53	Threshold	Dynamic
22	95.3%	34.05	6.03	96.7%	34.42	5.98	94.0%	33.68	6.08	Threshold	Threshold
23	97.8%	60.68	10.39	98.6%	70.71	12.11	97.0%	50.66	8.68	Threshold	Threshold
24	97.8%	63.84	10.84	98.7%	65.66	10.92	97.0%	62.02	10.77	Threshold	Threshold
25	93.7%	56.25	9.77	94.4%	60.82	10.88	93.1%	51.68	8.65	Threshold	Threshold
26	96.7%	48.66	8.49	97.4%	48.14	8.19	96.0%	49.19	8.78	Dynamic	Dynamic
27	97.1%	61.64	10.11	96.2%	57.99	9.38	98.0%	65.30	10.84	Dynamic	Dynamic
28	99.3%	65.83	11.12	100.0%	66.18	11.03	98.6%	65.49	11.22	Threshold	Dynamic
29	89.8%	60.45	11.29	94.2%	67.38	12.42	85.4%	53.51	10.16	Threshold	Threshold
30	100.0%	77.16	12.86	100.0%	76.00	12.67	100.0%	78.32	13.05	Dynamic	Threshold
31	97.3%	49.66	8.47	97.4%	53.12	8.69	97.1%	46.21	8.26	Threshold	Threshold
32	97.9%	29.76	5.13	97.3%	30.23	5.29	98.6%	29.30	4.96	Threshold	Threshold
33	99.4%	74.18	12.51	100.0%	76.90	12.82	98.7%	71.45	12.21	Threshold	Dynamic
34	94.3%	61.98	10.69	98.7%	70.53	12.05	89.8%	53.44	9.34	Threshold	Dynamic
35	98.8%	58.27	9.88	100.0%	61.16	10.19	97.6%	55.39	9.56	Threshold	Threshold
36	97.2%	63.73	10.76	97.0%	66.41	11.50	97.4%	61.06	10.02	Threshold	Threshold
37	93.1%	55.60	10.13	93.6%	57.66	10.49	92.5%	53.54	9.78	Threshold	Threshold
38	89.0%	33.03	5.89	94.6%	41.24	7.11	83.4%	24.82	4.68	Threshold	Threshold
39	94.2%	37.92	6.36	92.1%	46.43	7.78	96.3%	29.42	4.95	Threshold	Threshold
40	92.9%	41.45	7.18	85.8%	33.81	6.18	100.0%	49.08	8.18	Dynamic	Threshold
41	98.1%	53.07	8.87	98.7%	56.13	9.33	97.5%	50.00	8.41	Threshold	Dynamic
42	96.4%	63.26	10.82	94.3%	57.51	9.83	98.6%	69.02	11.81	Dynamic	Threshold
43	95.2%	61.49	10.61	90.4%	50.96	9.22	100.0%	72.03	12.00	Dynamic	Dynamic
44	98.6%	51.68	8.85	98.6%	50.87	8.71	98.6%	52.48	8.98	Dynamic	Threshold
45	95.8%	53.37	9.48	97.3%	60.62	10.44	94.2%	46.11	8.51	Threshold	Threshold
46	93.8%	50.61	8.81	91.3%	44.30	8.08	96.2%	56.93	9.55	Dynamic	Dynamic
47	100.0%	66.12	11.02	100.0%	66.77	11.13	100.0%	65.47	10.91	Threshold	Dynamic
48	100.0%	39.20	6.53	100.0%	37.87	6.31	100.0%	40.54	6.76	Dynamic	Threshold

**Table 3:** The time, accuracy, ITR, and OCM averages are displayed both study-wide and per interface for each spelling task.

	Threshold bar Interface Averages				Dynamic Interface Averages			
AVERAGE:	Time	Acc	ITR	OCM	Time	Acc	ITR	OCM
**ALL:**	41.82	95.71%	**54.59**	9.41	44.64	95.81%	**52.35**	9.04
**BCI:**	21.53	94.26%	**52.93**	9.17	23.64	96.09%	**53.69**	9.10
**HAVE_FUN:**	56.76	95.51%	**54.07**	9.49	56.94	95.79%	**52.34**	9.19
**PROGRAM:**	47.37	97.32%	**56.70**	9.57	53.41	95.54%	**51.04**	8.85

**Table 4:** Comparison of top quartile (top 25%) spelling task performances for dynamic and threshold bar interfaces in terms of accuracy, information transfer rate, and output characters per minute.

	Accuracy	ITR	OCM
Dynamic Q1	0.999	73.51	12.28
Threshold bar Q1	0.998	73.47	12.30

**Table 5:** Comparison of performance metrics between the study-wide average and the best-performing interface. The table shows potential gains in performance if the best interface had been chosen, highlighting the differences and percentage gains.

	Study Avg	Best Interface Avg	Difference	% Gain
ACC	96.00%	97.48%	1.48%	1.54%
ITR	53.61	58.00	4.39	8.18%
OCM	9.25	9.95	0.70	7.59%

## Data Availability

The availability of these data is restricted. Data were stored anonymously for analysis purposes only to ensure participant confidentiality. Ethical approval did not cover the publication of EEG data, so these data cannot be shared.

## Abbreviations

The following abbreviations are used in this manuscript:

BCI	Brain–Computer Interface
cVEP	Code-Modulated Visual Evoked Potential
EEG	Electroencephalogram
SSVEP	Steady-State Visual Evoked Potential
GUI	Graphical User Interface
ITR	Information Transfer Rate
OCM	Output Characters per Minute
VEP	Visual Evoked Potentials
IQR	Interquartile Range
SD	Standard Deviation
Hz	Hertz
bpm	Bits per Minute
s	Seconds
K	Number of Targets
C	Classifier Output
W	Wilcoxon Signed-Rank Test Statistic
Fs	Sampling Rate
CCA	Canonical Correlation Analysis
